# The Hospital Problem

**Published:** 1890-03-22

**Authors:** Henry C. Burdett

**Affiliations:** Deputy Chairman of The Hospitals Association


### The Hospital Problem.

By Mr. Henry C. Burdett, Deputy Chairman of The Hospitals Association.

*(Continued from page 371.)*

### III.—HOSPITAL AUDIT AND BOOK-KEEPING.

I now come to another important question—the audit of hospital accounts. I am strongly of opinion that an audit by a firm of accountants alone is not sufficient. I believe the best system to be that which makes the professional auditors responsible for the exact accuracy of the figures published, whilst associated with them should be one of the most active of the managers, who is enabled by his knowledge to go through every item, and to see that the statements are so arranged that nothing is suppressed or hidden which ought to be plainly stated. A combined system of audit where a firm of professional accountants is associated with one of the hospital governors is the one best calculated to secure the maximum of good management and economy.

### Hospital Bookkeeping and Accounts.

I venture now to briefly state the best system upon which hospital accounts can be kept. Every Report should contain the following accounts:—

(1) An Income and Expenditure Account, containing a detailed statement of the receipts and expenditure under classified heads;

(2) An Invested Property Account, showing all the property of the institution, the various securities held, and the income derived therefrom;

(3) A Balance-sheet;

(4) A Special Appeal Account. This should show all the money received as the result of appeals and personal canvassing, apart from old subscriptions, the Hospital Sunday and Saturday Funds, and other regular and assured sources of income. It should also show every item of expenditure connected with the issue of these appeals, including advertisements, salaries, commissions, printing, stationery, postage, and every other item of the kind. Such an account enables any governor to keep an eye upon the management, and to ascertain if the efforts put forth are adequate to the purpose, and if they combine the minimum of expenditure with the maximum of results.

If there are any special funds such as a Samaritan Fund, a Convalescent Fund, a Chaplain's Fund, and so forth, a separate statement should appear in the Report in each case.

Turning now to the books which it is desirable to keep, I may deal with them under two heads.

I. Receipts:—These should include (1) a Cash Book, (2) a Cash Analysis Book, (3) a Subscribers' Register, (4) a Legacy Book, and (5) an Invested Property and Rent Book.

The Cash Analysis Book will contain under their proper heads, every item of receipt throughout the year, and the total of each column, *i.e.*, donations, subscriptions, investments, and so forth, should agree with the total given in the published accounts as well as with the totals given at the end of the lists of subscriptions, donations, legacies, and invested property published in the Report. This is easily arranged by having two columns in the Report, one showing the amount of former subscriptions and donations received from individuals, and the other the amounts received from each during the past twelve months.

II. Expenditure. The books required to keep a correct account of the expenditure of public institutions are: (1) the Analysis Journals; (2) a Journal; (3) a Ledger; (4) a Wages Book; and (5) Petty Cash-books —*i.e.*, at least one for the secretary and one for the matron or lady superintendent.

I am free to confess that during the last twenty years I have made a large demand upon the patience, kindness, and consideration of the officers of the various hospitals throughout the country. I have often had to trouble them with inquiries and various forms to fill in, involving frequently a large amount of time and the exercise of no little patience and care. I am happy to take this opportunity of publicly thanking the whole of the officials all over the country for their generous co-operation and courtesy throughout this period. I have, of course, always been ready to assist anybody with information or facts when application has been made to me, and so I am proud to think a feeling of confidence has grown up between myself and the officials which enables us to trust and help each other to an extent which I believe has been fruitful in results to the benefit of the charities in which we are all so greatly interested. I should like to make some definite return for all the kindness I have received. Many of the inquiries and most of the returns would have been unnecessary had the accounts, of the larger institutions at any rate, been kept upon something like an identical plan. Unfortunately, where the managers have endeavoured to follow a particular form of accounts they have naturally adopted their own method of classification, and so the attempt has largely failed to accomplish what is desired. For instance, it is a common thing to find alcohol—*i.e.*, wine and malt liquors—placed in one report under "Provisions"; in another, under "Surgery and Dispensary"; and, in a third, under a separate heading of its own. Very many other items are treated in an equal variety of ways, and so the reports are most difficult to analyse, and it is almost impossible for any-one, even with the largest experience and knowledge, to compare the expenditure of one institution with that of another upon an identical basis. I have therefore thought that I might make some little return for the kindness I have received if I published a Glossary as an appendix to the system of accounts which I am about to explain to you, and the heads of which I have already given. I have this Glossary here on the table before me. It has been compiled with commendable diligence by Mr. Michelli, the secretary of the Seamen's Hospital at Greenwich, and I hope that in the course of the evening some of you will put it to the test by asking through the Chairman under what head particular items are to be classified. I have also arranged that leaves from the various books, together with this Glossary and a brief explanation of the system, shall be given in the forthcoming edition of the *Hospital Annual*, so that every person who is interested in the matter may be able to study them at leisure, and, if they please, to alter their system of accounts, and so obviate the necessity for many, if not for all, the inquiries so frequently addressed to them under existing circumstances. Such a general system of accounts, if generally adopted, must tend to secure to the officials no small amount of comfort and advantage.

### The Income and Expenditure of Individual Charities.

Finally I should like to say that I have prepared an analysis of the accounts of all the chief hospitals throughout the country. I have analysed them under various heads upon an identical basis, the whole of which will appear in the *Annual*. I should be very glad to answer any questions or to give the facts in regard to any particular institution, if anyone present who is interested will kindly ask for the information they require. I say this because The Hospitals Association now includes amongst its members representatives of hospitals in all parts of the country, and I think it not improbable that some of those present may desire to see how their particular institution stands when compared with the specimen cases which I have given in the course of this paper. It was not of course possible to include the whole on the present occasion, and so I have been obliged to confine myself to a few institutions, selected because they have the relatively largest or smallest expenditure, and so constitute a basis of comparison which all may profitably consider and study.

